# Prevalence and risk factors of deep vein thrombosis in patients after spine surgery: a retrospective case-cohort study

**DOI:** 10.1038/srep11834

**Published:** 2015-07-02

**Authors:** Si-Dong Yang, Huan Liu, Ya-Peng Sun, Da-Long Yang, Yong Shen, Shi-Qing Feng, Feng-Dong Zhao, Wen-Yuan Ding

**Affiliations:** 1 Department of Spinal Surgery, The Third Hospital of Hebei Medical University, 139Ziqiang Road, Shijiazhuang 050051, PR China; 2 Department of Orthopaedic Surgery, Tianjin Medical University General Hospital, 154 Anshan Road, Tianjin 300052, PR China; 3 Department of Orthopaedic Surgery, Sir Run Run Shaw Hospital, Zhejiang University School of Medicine, 3East Qingchun Road, Hangzhou 310016, PR China; 4 Hebei Provincial Key Laboratory of Orthopaedic Biomechanics, 139Ziqiang Road, Shijiazhuang 050051, PR China

## Abstract

Deep vein thrombosis (DVT) is common seen in patients undergoing spine surgery. However, its prevalence and associated risk factors have not been well understood yet. This retrospective case-cohort study was designed to investigate risk factors for postoperative DVT using retrospectively collected data from department of spine surgery between 07/2013 and 07/2014. Univariate analysis and binary logistic regression analysis were used to determine risk factors for DVT. A total of 861 patients were admitted into DVT-associated analyses, including 410 males and 451 females, aged from 15 to 87 years old (median 54, IQR 18). Of them, 147 cases (17%) sustained postoperative DVT. DVT incidence was 15.9% in patients undergoing lumbar interbody fusion, 13.5% in patients treated by low-molecular-weight heparin (LMWH), while only 8.1% in patients without LMWH. However, it revealed no significant difference between LMWH group and non-LMWH group (*χ*^2^ = 1.933, *p* = 0.164). Logistic regression equation was logit P = −4.09 + 0.05*X_1_ − 0.55*X_2_ + 0.41*X_3_ + 1.41*X_7_, (X_1_ = age; X_2_ = regions; X_3_ = hypertension; X_7_ = D-dimer). In this study, LMWH prophylaxis after spine surgery proved ineffective. Advanced age, D-dimer and hypertension have proved to be the risk factors for postoperative DVT in patients undergoing spine surgery.

Venous thromboembolism (VTE) is a common and potentially lethal disease that includes both deep vein thrombosis (DVT) and pulmonary embolism (PE)^1^. It can lead to severe morbidity with poor quality of life and even sudden death related to PE. Approximately half of all untreated DVT cases are complicated by PE, and conversely, 50% to 80% of all untreated PE cases are associated with DVT^2^,^3^. VTE has been known to be associated with advanced age, smoking, obesity, major surgery, hospitalization, immobilization, neurological deficit, blood transfusion, malignancy, trauma, inherited hypercoagulable state, and oral contraceptive use^4^,^5^,^6^. In spine surgery, the factors for venous stasis are considered to be long time horizontal ventral decubitus, lack of muscle tone, venous compression by retractors and postoperative bed rest. Venous intimal injury may occur in surgical handling^7^. VTE risk factors are common in patients with degenerative spine, and without prophylaxis, approximately 15% of patients undergoing posterior spinal surgery develop DVT^8^. Moreover, it has been reported that postoperative D-dimer assay can effectively predict DVT occurrence, and D-dimer level more than or equal to 500 μg/L is considered as a risk factor for DVT after spinal surgery^9^.

Most of the literature and international guidelines on VTE emphasize that prevention is more important and cost-effective than treatment, because once VTE develops, it can only be cured at considerable expense. Although approximately two-thirds of all VTE events result from hospitalization, only one-third of all hospitalized patients at risk receive adequate prophylactic treatment^10^. Currently, common prophylactic treatment mainly includes two types of therapies. One is mechanical prophylaxis providing intermittent pneumatic compression, and the other is pharmacological prophylaxis, mainly including low-molecular-weight heparin (LMWH)^11^. However, only a few studies have reported the effect of LMWH prophylaxis with controversial conclusions^4^,^12^,^13^.

Although DVT is common seen in patients undergoing spine surgery, its prevalence and associated risk factors have not been well understood yet. Therefore, this retrospective case-cohort study is designed to investigate the prevalence of DVT and explore the risk factors associated with DVT in the patients after spine surgery. The secondary goal is to assess the effect of LMWH prophylaxis in patients undergoing degenerative spine surgery.

## Patients and methods

### Ethics statement

The current study was approved by Ethics Committee of the Third Hospital of Hebei Medical University, also known as Ethics Committee of Hebei Provincial Orthopedic Hospital. There is no need to obtain informed consent from patients since this is a retrospective study and all the data were collected and analyzed anonymously. Also, it was approved by Ethics Committee of the Third Hospital of Hebei Medical University, also known as Ethics Committee of Hebei Provincial Orthopedic Hospital.

### Patients

This study retrospectively included patients who underwent spinal operations, admitted into Department of Spinal Surgery, The Third Hospital of Hebei Medical University in China, between 07/2013 and 07/2014. The inclusion criteria were complete medical records including patient number, sex, age, body weight, body height, regional distribution, admission date, hospital stay, occupation, lower extremity ultrasonography, DVT, spinal epidural hematoma, hypertension, diabetes, heart disease, surgical method, level and number of vertebrae fusion, duration of operation, blood loss, blood transfusion, length of incision, LMWH, prothrombin time activity (PTA), fibrinogen (FIB), thrombin time (TT), D-dimer, HDL (high density lipoprotein), LDL (low density lipoprotein), total cholesterol (TC), total bilirubin, direct bilirubin and indirect bilirubin. However, some loss of the above records were accepted to include more patients. The exclusion criteria were conservative treatment and percutaneous vertebroplasty, since the patients were discharged soon from hospital after percutaneous vertebroplasty. In addition, the patients were excluded if they suffered from pre-operation DVT, or ever took anticoagulant such as warfarin, aspirin and clopidogrel during one week before hospital admission.

## Methods

The methods were carried out in accordance with the approved guidelines. Three authors (HL, YS and DLY) identified and collected all the data of patients according to inclusion creteria and exclusion criteria. Another two authors (SDY and YPS) were responsible for data input using Microsoft Office Excel. In addition, three authors (SDY, FDZ and SQF) were responsible for data analyses. Statistical analyses were performed using SPSS for Windows, version 18.0 (SPSS Inc., USA). All measurement data are presented as the mean ± SD (standard deviation) when data satisfied criteria for normality with *p* > 0.10. Otherwise, it should be presented as median (interquartile range, IQR). When data satisfied criteria for normality and homogeneity of variance, statistical analysis between groups was performed using independent samples t-test. Otherwise, statistical analysis was performed using Mann-Whitney U test. For count data, it was presented as percentage, and chi-square test was used for data analysis. Non-conditional binary logistic regression model was used to explore the associated risk factors of DVT in the patients after spinal surgery. Values for *p* < 0.05 were regarded as significant with two-tailed tests.

## Results

### General data of patients included

These criteria were met in 1628 cases, which were therefore incorporated into this single-center retrospective cohort study (see supplementary dataset). Of the 1628 patients initially identified, a total of 861 patients were admitted into DVT-associated analyses and 767 were out for data deficiency of DVT records. All of the 861 patients were examined by lower extremity ultrasonography pre- and postoperatively, and subfascial drains were routinely used. The 861 patients included 410 males and 451 females, aged from 15 to 87 years old (median 54, IQR 18), 584 cases from rural areas and 277 from urban areas. Average hospital stay was 16 ± 5 days. Of them, 147 cases (17%) developed postoperative DVT, no PE cases, only 2 cases (0.23%) with spinal epidural hematoma, 714 cases (83%) without postoperative DVT.

### Comparison of age, gender, and regions

General information of the 147 postoperative DVT patients was shown in Table 1. Of them, 67 cases were males, 80 females, 108 cases from rural areas and 39 from urban areas. Of the non-DVT cases (714 cases), 343 cases were males, 371 females, 476 cases from rural areas and 238 from urban areas. Chi-square test indicated that there were no statistical differences regarding gender and region distribution between DVT group and non-DVT group (*χ*^2^ = 0.296, *p* = 0.586; *χ*^2^ = 2.585, *p* = 0.108, respectively). Age of the DVT group was 59(IQR 11) years old, the non-DVT group 51.5(IQR 18) years old. Mann-Whitney U test showed that DVT group was significantly older than non-DVT group (Z = −7.018, *p* < 0.001).

### Comparison of surgical data

The surgical data included surgical duration, blood loss, blood transfusion and incision length according to patient operation note. As shown in Table 2, blood transfusion in DVT group was significantly more than that in non-DVT group (*p* = 0.025). Also, incision length in DVT group was significantly longer than that in non-DVT group (*p* = 0.039). However, surgical duration and blood loss between the two groups were not statistically different (*p* > 0.05).

### Chronic disease history

As shown in Table 3, chronic disease history was compared between DVT group and non-DVT group, including high blood pressure (HBP), diabetes mellitus (DM) and heart disease (HD). It is noted that HBP in DVT group was more than that in non-DVT group (*p* < 0.001), while DM and HD between the two groups were no difference.

### Biochemical analyses

As shown in Table 4, biochemical analyses associated with postoperative DVT were conducted between DVT group and non-DVT group. Of them, FIB, D-dimer and HDL were found significant difference between the two groups (*p* = 0.022, *p* < 0.0001, *p *= 0.002, respectively), while the other items including PTA were of no significance (all *p* > 0.05).

### Hospital stay and BMI

Average hospital stay in DVT group was 17(5) days, and in non-DVT group 15(3) days. The difference between them was statistically significant (Mann-Whitney U test, Z = −5.387, *p* < 0.001). In addition, BMI(body mass index) was calculated in 89 cases, including 17 cases in DVT group and 72 in non-DVT group. BMI in DVT group was 25.83(2.98) and in non-DVT group 25.95(3.96), without significant difference (Mann-Whitney U test, Z = −0.898, *p* = 0.369).

### LMWH and DVT

In total, 721 cases were injected LMWH (4100UI/day) to prevent postoperative DVT, from the 1st postoperative day to the 7th postoperative day. Mechanical prophylaxis was used throughout admission. Of them, 97 cases sustained DVT and 624 did not; thus, DVT incidence was 13.5%. Meanwhile, 86 cases were not administrated LMWH after spine surgery, including 7 DVT cases and 79 non-DVT cases; thus, DVT incidence was 8.1%. Totally, DVT incidence was calculated to be 12.9%. Chi-square test between LMWH group and non-LMWH group revealed no significant difference regarding the use of LMWH after spine surgery (*χ*^2^ = 1.933, *p* = 0.164).

### Lumbar interbody fusion and DVT

In this study, 784 cases underwent lumbar interbody fusion. Of them, 575 cases were performed single level including 79 DVT cases, 178 cases with double levels including 38 DVT cases, and 31 cases with three levels or above including 8 DVT cases. Totally, DVT incidence in the patients undergoing lumbar interbody fusion was 15.9%. Chi-square test revealed that DVT incidence was significantly different in regards to fusion levels (*χ*^2^ = 8.215, *p* = 0.016). Furthermore, linear-by-linear association test (*χ*^2^ = 8.073, *p *= 0.004) showed significance regarding DVT incidence in single level (13.7%), double levels (21.3%), and three levels or above (25.8%).

### Logistic regression analysis

In this analysis, all patients were grouped into four categories according to their age (~45; 45 ~ 54; 55 ~ 64; 65~), and fusion levels were 0, 1, 2, 3~. The regression analysis method was set to be Backward(LR), probability for stepwise(Entry 0.10, Removal 0.15). As shown in Table 5, binary logistic regression revealed that age and D-dimer were risk factors for postoperative DVT, while regional distribution was likely a protective factor for postoperative DVT. Besides, HBP entered the regression equation. As a consequence, the logistic regression equation was presented as logit P = −4.09 + 0.05*X_1_ − 0.55*X_2_ + 0.41*X_3_ + 1.41*X_7_, (X_1_ = age; X_2_ = regions; X_3_ = HBP; X_7_ = D-dimer). The equation was statistically significant by Pearson Chi-Square Test (*χ*^2^ = 70.151, P < 0.0001).

## Discussion

Considering so many data are presented in this study, a summary of main findings are as follows. A total of 861 patients were admitted into DVT-associated analyses, including 410 males and 451 females, aged from 15 to 87 years old (median 54, IQR 18), 584 cases from rural areas and 277 from urban areas. Of them, 147 cases (17%) developed postoperative DVT, the other 714 cases (83%) not, no PE cases, only 2 cases (0.23%) with spinal epidural hematoma. DVT incidence was 15.9% in patients undergoing lumbar interbody fusion, and it was significantly different in regards to fusion levels (*χ*^2^ = 8.215, *p* = 0.016). Furthermore, linear-by-linear association test (*χ*^2^ = 8.073, *p* = 0.004) showed significant difference regarding DVT incidence in single level (13.7%), double levels (21.3%), and three levels or above (25.8%). DVT incidence was 13.5% in patients treated by LMWH, whereas it was only 8.1% in patients without LMWH prophylaxis. However, it revealed no significant difference between LMWH group and non-LMWH group (*χ*^2^ = 1.933, *p* = 0.164). Univariate analysis found that advanced age, blood transfusion, number of levels treated, FIB, D-dimer, HDL and hypertension tended to be risk factors for DVT in patients undergoing spine surgery. However, non-conditional logistic regression indicated that advanced age, hypertension and D-dimer were risk factors while regional distribution was protective factor for postoperative DVT, with the following equation logit P = −4.09 + 0.05*X_1_ −0.55*X_2_ + 0.41*X_3_ + 1.41*X_7_, (X_1_ = age; X_2_ = region, rural area = 0, urban area = 1; X_3_ = hypertension; X_7_ = D-dimer). Average hospital stay in DVT group was 17(5) days, more than that in non-DVT group with 15(3) days (*p* < 0.001).

In this study, it was found that increasing age was a risk factor for postoperative DVT after spine surgery, almost consistent with the other reports^4^,^14^. However, the included patients in the study of Strom RG *et al.* were much older with the mean age of 64 years old^4^, while in this study the age was from 15 to 87 years old (median 54, IQR 18). Nevertheless, that could not be the reason why the postoperative DVT incidence reported in the two studies was great different from each other. VTE incidence after spine surgery was reported only 4.3%, including acute/chronic DVT and PE in the study^4^. Inconsistent with what reported by Strom RG *et al.*, total DVT incidence after spine surgery reported in our work was 17%. In addition, DVT incidence was found to be 15.9% in patients undergoing lumbar interbody fusion, 22% in patients undergoing multi-level lumbar interbody fusion, and 13.5% in patients prophylactically treated by LMWH. Anyhow, DVT incidence reported in our study was much higher than that reported by Strom RG *et al.*

The reason may be the dosage or the duration of LMWH administrated to the patients? Strom RG *et al.* reported that the patients were routinely administered daily prophylactic enoxaparin at 40 mg for normal renal function, 30 mg for creatinine clearance of <30 mL/min, starting on the 1st postoperative day. And lower extremity ultrasonography was performed on patients with a sign or symptom of DVT (unilateral calf pain, edema, erythema, warmth) and those not mobilized by the 3rd postoperative day. Different from those, dosage of LMWH administrated to the patients was 4100UI daily in our study from the 1st postoperative day to the 7th postoperative day. Also, lower extremity ultrasonography was routinely performed on patients on the 7th day after spine surgery, because the patients were routinely advised by doctors to walk on foot after the 7th postoperative day. Thus, that could be some reasons for high DVT incidence in our study. It has been reported that DVT incidence of symptomatic patients is around 0.5% to 2.5% of the procedures in the spine, but it is estimated that the DVT incidence of asymptomatic patients exceeds 15%^15^, which is in line with the 17% DVT incidence found in our study.

In this study, 91.1% (784) of included patients underwent lumbar interbody fusion, confirming that lumbar spine is the region of greatest vulnerability in the whole spine^16^. As well, lumbar spine surgery is more likely to be associated with postoperative DVT incidence. It revealed in this study that DVT incidence was significantly different regarding fusion levels (*χ*^2^ = 8.215, *p* = 0.016) in the patients undergoing lumbar interbody fusion. Furthermore, linear-by-linear association test (*χ*^2^ = 8.073, *p* = 0.004) showed significant difference regarding DVT incidence in single level (13.7%), double levels (21.3%), and three levels or above (25.8%). Thus, the findings above were consistent with the report of Strom RG *et al.*, demonstrating number of levels treated as one statistically significant predictor of acute VTE (P = 0.012)^4^.

Oda T *et al.* reported that it was without prophylaxis that approximately 15% of patients undergoing posterior spinal surgery developed DVT^8^. Conversely, it was found in our study that DVT incidence was still high (13.5%) even with aggressive prophylaxis using LMWH. And the reasons remain unclear to us. To make us surprised and confused, DVT incidence was found much lower (8.1%) in the patients without LMWH prophylaxis, which was a contradictory phenomenon to what we had thought.

A prospective analysis by Smith MD *et al.* reported that the overall clinical prevalence of thrombotic complications was only 0.9% (three complications in 317 patients) after major reconstructive operations on the spine^17^. However, it is not difficult to attain the important reasons for that low VTE incidence, that thigh-high stockings and sequential pneumatic compression of the lower extremities were used in all patients for prophylaxis against venous thrombosis. As compared to the current study, a conclusion could be drawn that combined application of mechanical prophylaxis such as thigh-high stockings and sequential pneumatic compression, was more effective than LMWH prophylaxis for DVT in the patients undergoing spine surgery.

The presented results claim that the incidence for DVT was higher in patients treated with LMWH than in patients not treated with LMWH. However, statistical analysis revealed no significant difference in DVT incidence regarding the use of LMWH after spine surgery (*χ*^2^ = 1.933, *p* = 0.164). As for possible confounders, we have to admit its existence. The major confounder resulted from the two patient group compared in the analysis. LMWH group is mainly composed of the patients with lumbar fusion surgery, whereas non-LMWH group is mainly composed of those with ACDF. As mentioned above, the patients with lumbar spine fusion surgery always stay in bed for a week, while the patients with ACDF can walk on foot just on the first postoperative day. That is why our doctors make different decisions on different patients regarding the use of LMWH. Therefore, that is perhaps the reason for such high incidence of DVT in LMWH group, as compared to non-LMWH group. In addition, of the 861 cases, 784 cases underwent lumbar interbody fusion, almost all of them with LMWH treatment. Thus, the bias resulting from that is very limited in the analysis.

After spinal fusion surgery, by convention, the patients are asked to stay on bed for 7 days according to our clinical experience for the following reasons. First, we think they need some time to recover from the surgical trauma, maybe because we doctors in China are relatively conservative compared to Western doctors. Second, the patients need to be treated due to the edema of nerveroots and other postoperative complications. However, during the period, the patients were not immobilized, but encouraged to do lower-limb exercise on bed to accelerate blood circulation during the on-bed period. Apparently, this treatment is not evidence-based medicine but our convention, and perhaps the reason for such high incidence of DVT. Surely, it should not be neglected when assessing the results presented in this study.

Most studies including ours were focused on DVT incidence in adult patients. However, it may be another case in the child patients regarding the DVT incidence. Recently, a retrospective review reported thromboembolic complications in children after spinal fusion surgery^18^. It was found that the incidence of VTE in children varied from 9.6 to 38.5 events per 10,000 spinal fusions (mean: 21 events per 10,000 spinal fusions) depending on the year, and the incidence of PE varied from 0 to 6 events per 10,000 spinal fusions (mean: 2 events per 10,000 spinal fusions); there were no in-hospital VTE-associated mortalities. A higher incidence of VTE in children was associated with older age and certain diagnoses (congenital scoliosis, syndromic scoliosis/kyphoscoliosis, and thoracolumbar fractures). As compared to the present study, DVT/VTE incidence in child patients is pretty lower than adult patients after spine surgery.

There is little data on when therapeutic anticoagulation may be safely initiated after spine surgery. Thus, there is no consensus on its role after degenerative spine surgery^19^. Postoperative hematoma has been reported due to heparinization^20^. A spinal epidural hematoma can cause profound neurological deficits, which has led many authors to advocate against pharmacological prophylaxis for routine spine surgery, particularly after decompressive laminectomy^21^. Therefore, some authors have advocated withholding anticoagulation for at least 1 week after surgery^20^,^22^. In our study, 2 cases (0.23%) developed spinal epidural hematoma in 3 days after spine fusion surgery. All of the patients in our study received LMWH prophylaxis from the 1st postoperative day to the 7th postoperative day. Also, subfascial drains were routinely used in all patients after surgery. Thus, it would be hard to determine the reasons for hematoma development, also hard to correlate hematoma with LMWH use.

While in this study it is possible that some thrombi may have escaped both clinical and ultrasonic detection, such thrombi apparently were not enough of a danger to warrant the use of intensive prophylactic procedures that are associated with more risk. On the basis of high DVT incidence found in this retrospective case-cohort study, therefore, we think that routine screening for the detection of DVT in patients who have had a procedure on the spine is warranted.

This is a retrospective case-cohort study on DVT prevalence and its risk factors in patients undergoing spine surgery. It was found that DVT incidence was high (17%) in patients undergoing spine surgery. Univariate analysis found that advanced age, blood transfusion, number of levels treated, FIB, D-dimer, HDL and hypertension tended to be risk factors for DVT. Logistic regression indicated that advanced age, hypertension and D-dimer were risk factors while urban area (vs rural area) was protective factor for postoperative DVT. On the basis of high DVT incidence found in this retrospective case-cohort study, therefore, we think that routine screening for the detection of DVT in patients undergoing spine surgery is warranted. Although Doppler ultrasonography of lower limbs has proved to be efficient to confirm existence of DVT, this study laid emphasis on associated risk factors for the purpose of early alert and prevention for DVT. Of note, HDL and hypertension were found for the first time as risk factors of DVT in patients undergoing spine surgery.

Overall, this study was able to detect some interesting correlations that will help understanding DVT development in patients undergoing spine surgery. Also, some findings in this study can be early alert for postoperative DVT development. However, the retrospective nature of our work goes along with limitations, the most obvious being the dependence upon the quality of the data recorded in the medical records. Furthermore, a selection bias cannot be excluded since so many patients were excluded for data deficiency. In fact, our hospital is the biggest orthopaedic hospital in Hebei Province; hence, it was unavoidable to incorporate relatively more acute and severe patients in this study.

In summary, in this case-cohort study, LMWH prophylaxis in the patients undergoing degenerative spine surgery proves to be ineffective. However, the confounders should not be neglected when assessing the results presented in this study. Additionally, advanced age, D-dimer and hypertension have proved to be the risk factors while urban area (vs rural area) be protective factor for postoperative DVT in the patients undergoing spine surgery.

## Additional Information

**How to cite this article**: Yang, S.-D. *et al.* Prevalence and risk factors of deep vein thrombosis in patients after spine surgery: a retrospective case-cohort study. *Sci. Rep.* **5**, 11834; doi: 10.1038/srep11834 (2015).

## Supplementary Material

Supplementary Information

## Figures and Tables

**Table 1:** General information of the 147 postoperative patients with DVT.

Items	Data in detail
Number of DVT	93 patients with single site; 40 with double sites; 14 with 3 or above
Distribution of DVT	DVT in calf muscular venous with 117 patients; others 30 patients
Diagnosis	Lumbar disc herniation (135 cases); cervical spondylopathy (11 cases); thoracic spinal stenosis (1 case)
Fusion and fixation?	Yes for 134 cases including 83 with single level, 41 with double levels and 10 with 3 levels or above

DVT, deep vein thrombosis.

**Table 2:** Comparison of surgical data associated with postoperative DVT.

Items	DVT group	Non-DVT group	Mann-Whitney U test	
	Patients/Median(IQR)	Patients/Median(IQR)	Z-value	*p*-value
surgical duration	147/170(80) min	713/160(65) min	−1.424	0.154
blood loss	147/500(500) ml	710/500(500) ml	−1.090	0.276
blood transfusion	65/400(400) ml	255/300(250) ml	−2.246	0.025
incision length	140/15(5.5) cm	700/16(6) cm	−2.064	0.039

DVT, deep vein thrombosis; IQR, interquartile range.

**Table 3:** Comparison of chronic disease history associated with postoperative DVT.

Items	DVT patients	Non-DVT patients	OR	Chi-square test	
	Yes/No	Yes/No		*χ*^2^-value	*p*-value
HBP	54/93	161/553	1.99	13.093	<0.001
DM	18/129	66/648	1.37	1.247	0.264
HD	7/140	34/680	1.00	Fisher’s exact test	1.000

DVT, deep vein thrombosis; OR, odds ratio; HBP, high blood pressure; DM, diabetes mellitus; HD, heart disease.

**Table 4:** Biochemical analyses associated with postoperative DVT.

Items	DVT group	Non-DVT group	Mann-Whitney U test	
	Patients/Median(IQR)	Patients/Median(IQR)	Z-value	*p*-value
FIB	146/2.96(0.59) g/L	706/2.81(0.82) g/L	−2.288	0.022
TT	146/14.6(1.5) s	706/14.6(1.7) s	−0.08	0.936
D-dimer	146/0.18(0.24) mg/L	705/0.12(0.10) mg/L	−6.601	<0.0001
HDL	146/1.19(0.43) mmol/L	702/1.11(0.36) mmol/L	−3.026	0.002
LDL	146/3.16(1.00) mmol/L	702/3.12(1.12) mmol/L	−0.55	0.583
TC	141/4.89(1.21) mmol/L	681/4.74(1.20) mmol/L	−1.782	0.075
T-BIL	141/12.90(6.45) umol/L	682/12.50(6.00) umol/L	−1.311	0.190
D-BIL	141/3.90(2.35) umol/L	682/3.80(2.10) umol/L	−1.434	0.152
I-BIL	141/9.20(4.60) umol/L	682/8.60(4.30) umol/L	−1.212	0.225

DVT, deep vein thrombosis; IQR, interquartile range; FIB, fibrinogen; TT, thrombin time; HDL, high density lipoprotein; LDL, low density lipoprotein; TC, total cholesterol; T-BIL, total bilirubin; D-BIL, direct bilirubin; I-BIL, indirect bilirubin.

**Table 5:** Binary logistic regression analysis of postoperative DVT.

NO.	Items	B	Exp(B)	*p*-value	95% CI for Exp(B)
X1	Age	0.05	1.05	0.000	(1.03, 1.07)
X2	Regions	−0.55	0.57	0.016	(0.37, 0.90)
X3	HBP	0.41	1.51	0.004	(1.09, 2.30)
X4	Fusion	0.19	1.21	0.167	(0.92, 1.59)
X5	Incision	−0.03	0.97	0.178	(0.93, 1.01)
X6	FIB	−0.01	0.99	0.908	(0.77, 1.26)
X7	D-dimer	1.41	4.09	0.000	(2.12, 7.88)
X8	HDL	0.13	1.14	0.288	(0.89, 1.46)
X9	TC	0.11	1.12	0.255	(0.92, 1.36)
X10	LMWH	0.82	2.28	0.298	(0.48, 10.73)
X0	Constant	−4.09	0.017	0.000	—

DVT, deep vein thrombosis; CI, confidence interval; HBP, high blood pressure; FIB, fibrinogen; HDL, high density lipoprotein; TC, total cholesterol; LMWH, low-molecular-weight heparin.
